# Cost-efficient management of peatland to enhance biodiversity in Finland

**DOI:** 10.1038/s41598-024-52964-x

**Published:** 2024-01-30

**Authors:** Parvez Rana, Priscillia Christiani, Anssi Ahtikoski, Soili Haikarainen, Leena Stenberg, Artti Juutinen, Anne Tolvanen

**Affiliations:** 1 Natural Resources Institute Finland (Luke), Oulu, Finland; 2 Natural Resources Institute Finland, Turku, Finland; 3 Natural Resources Institute Finland, Helsinki, Finland

**Keywords:** Environmental impact, Ecological modelling

## Abstract

Peatlands provide a variety of ecosystem services besides being important ecosystems for biodiversity. Sustainable peatland management requires that its impacts are identified, and all management is allocated in a cost-efficient manner. In this study, we assessed how peatland management influences the habitat suitability of red-listed plant species and the financial performance of management measured as net present value (NPV). The study was done in three landscapes in Finland. We considered four peatland management scenarios i.e., no management activity (NOMANAGE), hydrological restoration (REST), wood harvesting for bioenergy (BIOENERGY), and timber production (TIMBER). The NPVs of different management scenarios were compared to the habitat suitability of red-listed peatland plant species. A cost-impact analysis was used, with TIMBER as a baseline scenario, to find out which alternative scenario would be the most cost-efficient in contributing to habitat suitability. Our study shows that potential habitat areas were significantly different between the scenarios. REST provided the largest potential habitat areas, followed by BIOENERGY, NOMANAGE, and TIMBER. TIMBER provided the best financial performance when low interest rates were used. REST and BIOENERGY were more cost-efficient in enhancing potential habitat areas than NOMANAGE. REST would improve suitable habitats and provide financial benefits when a higher interest rate was used. In conclusion, even a *win*–*win* condition could be achieved in some cases (33%), in which higher NPV was achieved simultaneously with improved potential habitat areas. The study provides information for alleviating the economic barriers of restoration and targeting land use and management options cost-efficiently.

## Introduction

Peatlands play an important role in sustaining biodiversity and providing a variety of ecosystem services such as carbon storage, timber, recreation, natural products, and improving water quality^1–4^. Since the 1950s, approximately 15 million hectares of peatlands in boreal and temperate zones have been drained for various land uses, including forestry^5,6^. Drainage has caused the degradation of biodiversity^4,7^ and caused widespread decrease in the provision of peatland and freshwater ecosystem services^8,9^.

Peatland restoration aims at bringing back the biodiversity and ecosystem functions typical to pristine peatlands. One of its key ecological benefits lies in its potential to create suitable habitat areas for peatland plant species^10,11^. By focusing on plant species, restoration efforts not only contribute to the conservation of endangered plant life but also enhance the overall biodiversity of peatlands. As these plants flourish, they play a pivotal role in supporting the broader ecosystem by providing sustenance and habitat for various wildlife species^10,11^. There is evidence of successful development after restoration showed by field studies^12–24^ and simulation models^25–27^ although restoration may not return the peatlands fully to their pristine state^18,26,28^.

Efforts have been made through international policies to halt the degradation of peatlands and restore their biodiversity and ecosystem services. For example, the Convention on Biological Diversity^29^ and the EU Biodiversity Strategy to 2020^30^ established goals to combat biodiversity loss, such as aiming to restore 15% of degraded ecosystems by 2020. The targets were not achieved, and the EU member states failed to deliver a sound national restoration framework^31^. Therefore, the EU launched the Biodiversity Strategy for 2030^32^ and a proposal for a Nature Restoration Law, which sets out legally binding targets for biodiversity goals for 2030 and 2050^33^.

According to a cross-European study, the major barriers to restoration are socioeconomic and are especially related to the limited financial resources of public authorities and conflicting interests among stakeholders^34^. There is still a need to continue the management of peatlands to produce timber and raw material. For example, approximately 10 million hectares of peatland in Finland (which accounts for 27% of the country's total area, according to Tanneberger et al.^35^) contributed to 23% of the country's timber resources and 22% of annual tree growth (as reported by Korhonen et al.^36^). Additionally, in 2022, peatland in Finland served as the source of around 3% of the country's energy^37^. Timber harvesting is projected to increase from 60 to 80 million m^3^, largely from forested peatlands^38^. Hence, peatland restoration comes with a cost that is often questioned and compared with other alternative management scenarios, e.g., timber or bioenergy production, which are considered to be more profitable^39,40^. For wide-scale improvement of peatland biodiversity to occur, socioeconomic barriers must be overcome. To find a desirable solution from biodiversity and socioeconomic perspectives, alternative peatland management scenarios must be assessed for their contribution to improving peatland biodiversity in the most cost-efficient and socially acceptable ways^41–44^.

There is a large number of studies on peatland management and restoration, including, for example, studies on costs of restoration^45,46^, local peoples’ conflicting interests towards management and restoration^39,42,47,48^, and valuation of ecosystem services provided by peatlands^41,49–53^. Of previous studies, the closest to our research is Juutinen et al.^41^, which elaborated on tradeoffs between ecosystem services and biodiversity in drained peatlands by considering alternative land management scenarios and applying numeric optimization. Juutinen et al.^41^ found strong tradeoffs between biodiversity, greenhouse gas balances, and nutrient loading to watercourses over the period of 50 years in northern Finland. According to the tradeoffs identified, it is necessary to make compromises to achieve a landscape that is multifunctional and provides various ecosystem services in a cost-effective manner.

In this paper, we focused on peatland biodiversity. Our aim was to find peatland management alternatives that safeguard the peatland biodiversity in a cost-efficient way. As a proxy to biodiversity, we used our most recent model on the habitat suitability of red-listed plant species^54^ and elaborated the model to predict the habitat suitability over a 100-year time period. To improve the generalizability and applicability of our results, we include three landscapes of varying peatland types and areas from different parts of Finland in our assessment.

We compared four different management scenarios i.e., no management activity (NOMANAGE), hydrological restoration (REST), wood harvesting for bioenergy (BIOENERGY), and timber production (TIMBER), to find out which scenario would contribute the most towards biodiversity. We projected suitable habitat areas into different future conditions and alternative peatland management. We used net present value (NPV) to assess the financial performance of the scenarios. We employed a cost-impact analysis to explain further cost-efficiency, which involved expressing impacts in physical units while expressing costs in monetary terms^55^. The cost-impact analysis provides cost-efficiency measures for alternative management options so that their cost-efficiency can be ranked for decision-making.

Our main research questions were: (1) Do different management scenarios differ in terms of enhancing red-listed plant species suitable habitat areas? (2) What is the financial performance of different management scenarios? (3) What is the most cost-efficient way to improve suitable areas for red-listed plant species? The result of the study may help landowners and policymakers to alleviate the economic barriers of restoration and to target land use and management options cost-efficiently.

## Methodology

### Study regions

There are 10 million hectares (Mha) of peatlands in Finland, half of which have been drained for forestry purposes. Regarding wood-production potential, the area of productive peatlands is about 3.8 Mha, which is almost 83% of the total drained peatland area^56^. The remaining area is classified as low-productive peatlands (annual increment of growing stock over rotation  < 1 m^3^ha^−1^a^−1^), being mostly nutrient-poor or nutrient imbalance sites^57^.

**Figure 1:**
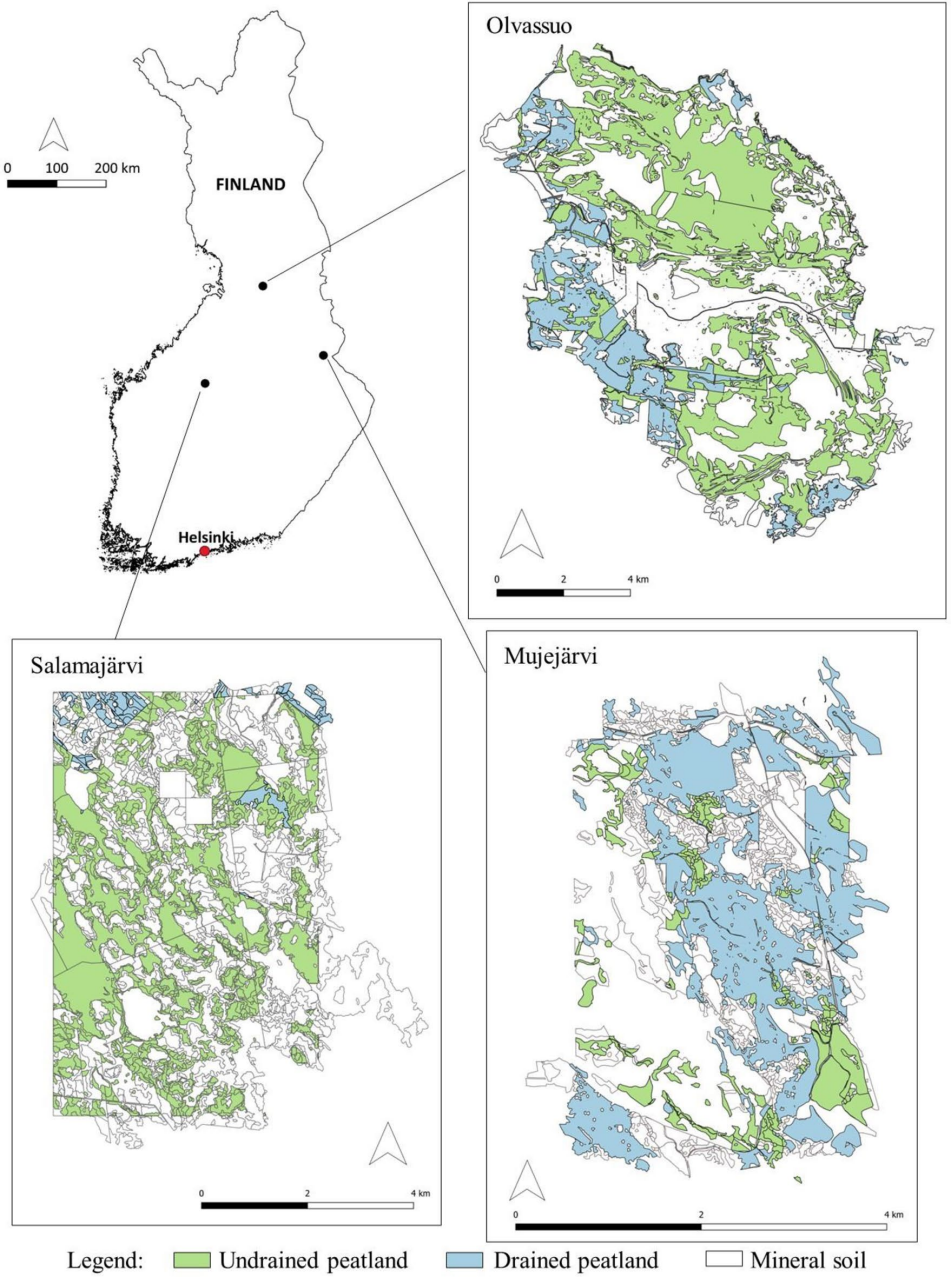
The geographical location of the study regions. Stand-wise data were derived from the databases of Metsähallitus (https://www.metsa.fi/en) and the Finnish Forest Center (https://www.metsakeskus.fi/en). The map was created using QGIS software (version 3.32.0, http://qgis.org).

The study was conducted in three case study regions: Salamajärvi, Mujejärvi, and Olvassuo in central Finland (Fig. 1). The total area without water in the study regions varied from 11 to 83 km^2^ (Table 1). The share of undrained and drained peatlands from the total land area of case study regions was 47% and 3% in Salamajärvi, 50% and 17% in Olvassuo, and 16% and 50% in Mujejärvi, respectively. The remaining share consisted of mineral soil stands. The rationale of combining peatland and mineral soil stands was to keep the study regions as entities by focusing on landscape level analysis. Practical forestry favors entities to avoid stand fragmentation and to keep operational costs in control. Stand-wise data (stand represents a forest management unit) were derived from the databases of Metsähallitus (state-owned forests; see https://www.metsa.fi/en) and the Finnish Forest Center (private-owned forests; see https://www.metsakeskus.fi/en) and represent the year 2021.

**Table 1:** Number of stands (N), total area, and the proportions of different types of sites in the study regions.

Region	N	Total area (km^2^)^a^	Proportion (%) of different types of sites							
			Protected^b^	Unprotected	Undrained peatland (productive)	Undrained peatland (low prod.^c^)	Drained peatland (prod.)	Drained peatland (low prod.)	Mineral soil (prod.)	Mineral soil (low prod.)
Salamajärvi	1899	42.8	73	27	19.7	27.0	2.8	0.6	49.7	0.1
Mujejärvi	1498	10.6	3	97	8.6	6.0	37.6	10.7	35.4	1.7
Olvassuo	3515	83.0	3	97	6.2	43.8	12.4	4.7	32.3	0.7

^a^Sum of the stands.

^b^Protected by the Natura 2000 program (European Commission, 2018).

^c^Low-productive stand: annual increment of growing stock over rotation  < 1 m^3^ha^−1^a^−1^.

### Management scenarios

First, in order to describe how alternative management of drained peatlands impact on the potential for suitable habitats of red-listed peatland plant species and financial performance in the study areas, four realistic management scenarios were considered: (i) NOMANAGE: no management activities, (ii) REST: hydrological restoration, (iii) BIOENERGY: wood harvesting for bioenergy, and (iv) TIMBER: practical forestry focusing on timber production. The first two, NOMANAGE and REST, were applied in both protected and unprotected areas, whereas the other two, BIOENERGY and TIMBER, were applied only in unprotected areas.

Then, the stand projections within the three regions, both peatland and mineral soil stands, were predicted by long-term simulations according to the four scenarios. The management scenarios consisted of detailed schedules on stand-level silvicultural activities concerning e.g., how and when to thin, to execute ditch network maintenance, or to apply final harvest. Undrained peatland stands were kept unmanaged, and mineral soil stands were treated according to the prevalent silvicultural guidelines^58^ across all scenarios. The management scenarios were applied as follows.

In NOMANAGE, all management activities, e.g., harvesting and ditch network maintenance, were forbidden in simulations concerning drained peatlands. It was anticipated that the stand would gradually become naturally rewetted and evolve into an ecosystem resembling undrained peatland^41,57^. There was no harvesting of timber on drained peatlands in this scenario, which means that the NPV of timber production from those sites was zero. (Note that NPV of mineral stands was positive in all scenarios.).

In REST, all trees on drained peatland stands were harvested except some trees that were left on the sites which have had trees before drainage. The ditches were blocked after harvesting, and it was assumed that the area would rewet rapidly, as happens under real situation^13^. The regeneration and growth of new trees after restoration was estimated according to the average growth of typical sites in the regions. Net revenue from harvested trees and restoration costs (800 € ha^−1^) were included in the NPV.

In BIOENERGY, almost all trees on drained peatland stands were simulated to be harvested. Approximately 20 trees per hectare were left as retention trees at sites where there had been trees before drainage. After harvesting, the natural regeneration of trees and the development of stands were simulated according to stand productivity (productive, low-productive) and site type (according to site fertility, characterized by dominant vegetation). As ditches were left untouched, the water table was expected to rise gradually due to the deterioration of ditches (see also Juutinen et al.^41^). NPV of timber production included net revenues from energy wood harvesting. Hence the landowners were expected to receive some income from tree harvesting, although active management was not continued.

In TIMBER, silvicultural activities could include pre-commercial and commercial thinnings, final harvest, regeneration activities, or fertilization, depending on the site type and the development stage of the stand. On productive drained peatlands, drainage was kept at a suitable level for tree growth, which means that if the evapotranspiration of trees was inadequate, ditch network maintenance was applied in the simulations. Stands were fertilized with wood ash (3–5 tons per ha). In low-productive peatlands, silvicultural activities were not applied due to the low profitability of those stands. Logging residues were not harvested. NPV of timber production included discounted net harvest revenues and silvicultural costs (Table S1).

### Potential habitat conditions analysis

We used our earlier habitat suitability models of Rana and Tolvanen^54^ on 33 red-listed plant species^59^ as a proxy for more general indications of regaining habitats by restoration for peatland biodiversity. The impact of each management scenario on red-listed plant species was assessed using Maximum Entropy modeling^60^. Potential habitat areas were first modeled to represent the present environmental condition and then projected into different future environmental conditions considering four management scenarios and extrapolating environmental condition of variables to new conditions up to 100 years.

A total of 16 environmental variables were used to predict the potential habitat areas in Rana and Tolvanen^54^ and we also used the same variables in this study, such as (1) growing degree days, (2) mean water balance (WAB), (3) topographic wetness index, (4) the proportion of calcareous rock, (5–8) tree species' mean volumes (pine, spruce, birch, and other deciduous species), (9–12) proportion of site type (herb-rich, *Vaccinium myrtillus*, *Vaccinium vitis-idaea*, and *Cladina*), (13) proportion of drained peatland area, (14) proportion of undrained peatland area, (15) proportion of open peatlands and (16) biodiversity index. Seven out of 16 environmental variables were simulated over the next 100 years (see Section "Simulations of environmental variables"), and the remaining nine variables were kept unchanged in the habitat suitability analysis. We selected these seven variables (mean volume of pine, spruce, birch, and other deciduous tree species, WAB, and the proportions of undrained and drained areas) based on availability in the simulation results and the contribution in the habitat model of Rana and Tolvanen^54^.

In the end, we calculated the total potential habitat area for red-listed species as a performance index for each management scenario. We compared the difference in the suitable area between the four scenarios using 95% confidence limits.

#### Simulations of environmental variables

Environmental variables were simulated for each management scenario. Four variables related to tree species volumes were simulated using the Motti simulator (see details below), whereas WAB was simulated using the SUSI simulator, and the proportions of drained and undrained peatland areas were estimated using a simple linear assumption.

The tree species' mean volumes needed in the habitat suitability analysis were based on the stand-wise simulations of the development of forest stands in the study regions. The input data for simulations was the initial forest data, i.e., altogether ca. 7000 stands (Table 1), and the growth and yield of each stand were predicted for 100 years according to management scenarios i–iv. In the stand projections, the Motti simulator, a tool to forecast growth and yield at the stand level, was used. Motti is efficient in assessing the impacts of alternative forest management practices on stand dynamics and forest management profitability and comparing different management strategies^61,62^. Motti includes a large set of stand-level and tree-level models to predict stand dynamics (regeneration, growth, and mortality) in different stages of stand development and in different circumstances (i.e., site fertilities, climatic conditions, and with or without silvicultural treatments^63^. Separate models are available for mineral soil and peatland stands (e.g., Refs.^64,65^). The technical details of Motti are described in Salminen et al.^61^.

WAB was predicted for 100 years using the SUSI simulator^66^. SUSI is a tool to model hydrology and stand growth for drained peatlands. It takes into account growth limiting factors under different land use schemes, site types, and site conditions. SUSI uses daily weather forcing, ditch depth, strip width, peat properties, and forest stand characteristics as input^67^. When simulating WAB using SUSI, silvicultural treatments and stand growth defined by Motti was used to ensure that WAB was in line with other variables used in the habitat suitability modeling. In SUSI, specific changes for ditch spacing and depth were simulated for each scenario (Table S2). As the ditches tend to deteriorate over time and lose their drainage capacity^68,69^, the depth of ditches was simulated to change gradually in the NOMANAGE, BIOENERGY, and TIMBER scenarios. The temperature and precipitation data for the three study areas were acquired from Finnish Meteorological Institute’s 10 km × 10 km grid data^70^. The current climate was represented by a 30-year period (1984–2013) that was repeated for 100 years.

The proportion of drained and undrained peatland area in the simulations was calculated based on the ditches' condition during the simulated 100 years. For NOMANAGE and BIOENERGY, we created the proportion of drained peatland area based on a simple linear development assumption. At the beginning of simulations (0-year development), drainage data from the Finnish Environment Institute (2009) was used to estimate the present proportion of drained peatland area. Since the ditches deteriorate gradually over time, the percentage of drained areas will decrease following the assumption that the drained area will slowly change into the undrained area. At the end (100th-year of development), we assumed that only 30% of the drained area remains in NOMANAGE and BIOENERGY. The linear decrease in drained proportion was calculated as the increase in undrained area in NOMANAGE and BIOENERGY. For REST, we assumed that the drained area proportion would be 0% in all drained areas throughout the 100 years simulation period. This assumption follows the purpose of the hydrological restoration to raise the water table. For TIMBER, we assumed the drained proportion would be at 100% throughout the 100 years simulation period since the areas were used for maximizing the production of timber (Table S3 and S4).

### Financial analysis

Along with the variables describing stand dynamics in different management scenarios, Motti simulator produced the economic variables for the financial analysis. In this study a Net Present Value, NPV calculation was applied. In brief, the NPV describes the capitalized value of land including all future net revenues which are discounted to present. The NPV combined here the costs (of silvicultural measures) and incomes (from thinnings and clear-cutting) for each individual stand in each management scenario, and further commensurate them by discounting (see, e.g., Ref.^71^ for the technical framework of NPV). We applied NPV instead of bare land value, BLV, since the time horizon for simulations in each forest management scenario was identical, 100 years (see Chang and Gadow^72^ for further details on the mathematical background of NPV vs. BLV). The NPV was calculated according to:

$$NPV_{a} = \mathop \sum \limits_{s = 1}^{S} \left[ {\mathop \sum \limits_{i = 0}^{T} b^{{t_{i} }} \left( {\mathop \sum \limits_{k = 1}^{K} CI_{ki}^{s} - \mathop \sum \limits_{m = 1}^{M} SC_{mi}^{s} } \right)} \right],$$

where NPV_a_ is the net present value for forest management scenario *a, a* = REST,…, NOMANAGE, BIOENERGY, TIMBER, *s* = an individual stand, *s* = 1,..,*S* total amount of stands depending on the study region (Salamajärvi, Mujejärvi or Olvassuo), *t*_*i*_ is a year within the time horizon *i* $\in$ {0,…100} *t*_*0*_ corresponding to calendar year 2021, *b* is the discount factor (*b* = 1/(1 + r) where *r* is the interest rate in real terms), *CI*_*ki*_ indicates cutting income from the *k*th thinning at year *t*_*i*,_ *s.t*. *K* indicates final harvesting, and SC_mi_ is a cost for a silvicultural action *m* at year *t*_*i*_. Notice that all removed trees are accounted for, also those removed during restoration in REST scenario. Notice further that in NOMANAGE scenario silvicultural actions and harvesting took place only on mineral soil stands while in other scenarios (REST, BIOENERGY and TIMBER) there were silvicultural actions and harvesting also on peatland stands.

In assessing the NPVs, we applied stumpage prices and silvicultural costs according to nominal time series covering the calendar years 2015–2019. This was the most recent 5-year time series for stumpage prices and silvicultural costs at the time of the analyses. The 5-year time series was selected to capture business cycles so that the calculated average values would include both peak and bottom of prices and costs. The original nominal time series of stumpage prices and silvicultural costs were deflated by the cost-of-living index (^73^; the base year 2019) to attain real prices and costs (Table S1).

### Cost-impact analysis

The NPVs of different management scenarios were compared to the potential habitat suitability area of red-listed peatland plant species as a cost-impact analysis. For comparison, TIMBER was chosen as the baseline scenario to be compared with other scenarios since TIMBER presents practical forestry focusing solely on timber production, and it ignores the provision of any other ecosystem service. In the comparison, the measure was the cost (in terms of NPV loss compared to TIMBER, € ha^−1^) of a scenario to result in an extra hectare area suitable for red-listed plant species. The rationale of cost-impact analysis was to find out which scenario would be the most cost-efficient in contributing to enhancing habitat suitability. We also investigated whether the interest rate (2–5%) would play a relevant role in cost-efficiency.

## Results

### Environmental variables during the simulations

**Figure 2:**
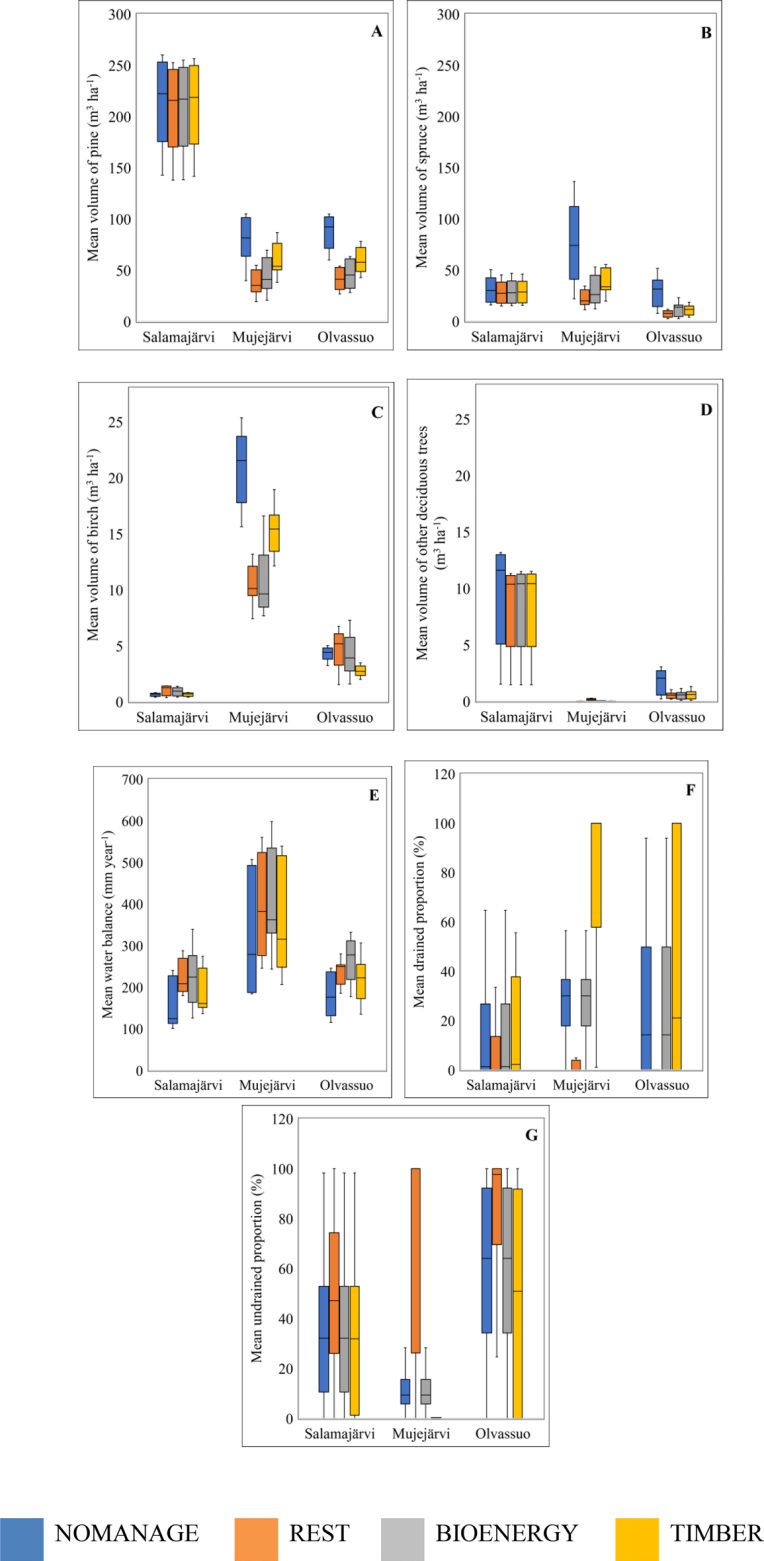
Simulated mean volumes for trees (**A**: pine, **B**: spruce, **C**: birch, **D**: other deciduous trees), water balance (**E**), mean proportion of drained peatlands (**F**), and mean proportion of undrained peatlands (**G**) for each management scenario across 100 years. A box shows the first quartile to the third quartile. The line inside the box represents the median, whereas the whisker shows the minimum and maximum values. Please note the different scaling between figures.

The mean value of tree species’ volumes obtained during 100-year simulations seemed to vary more between the case study regions than between the four management scenarios (Fig. 2). In general, Salamajärvi had the highest mean volumes of pine, and Mujejärvi had the highest volumes of birch. The NOMANAGE scenario produced the highest volumes of pine and spruce in Mujejärvi and Olvassuo and the highest volumes of birch in Mujejärvi. The volumes represented in Fig. 2 include all simulated stands in the regions. (i.e., undrained and drained peatlands and mineral soils). Notably, the scenarios differ from one another only in the drained peatlands.

The mean proportion of drained and undrained peatland areas obtained during 100 years of simulation varied between the case study regions and also between the management scenarios (Fig. 2). In general, Mujejärvi had the highest mean proportion of drained peatland areas, and Olvassuo had the highest mean proportion of undrained peatland areas. TIMBER scenario produced the highest mean proportion of drained peatland areas as the study area was continuously kept drained for timber production for all regions. REST scenario produced the highest mean proportion of undrained peatland areas because the restoration changed all drained areas into undrained areas.

### Suitable habitat area in different management scenarios

**Figure 3:**
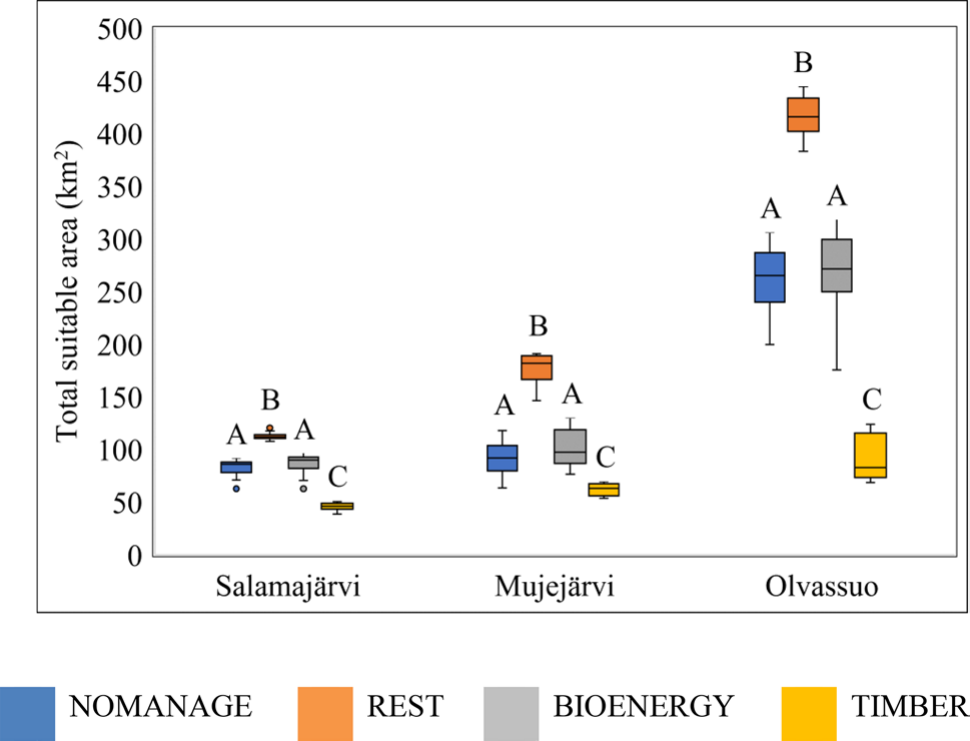
Total potential habitat area for studied plant species (33 species in Salamajärvi, 32 in Mujejärvi, 33 in Olvassuo) for each management scenario. The mean values were calculated across 100 years. A box shows the first quartile to the third quartile value. The line inside the box represents the median, whereas the whisker shows the minimum and maximum values. Bars with the same letters are not significantly different based on 95% confidence limits.

REST provided the largest potentially suitable habitat area, followed by BIOENERGY and NOMANAGE, and at last, TIMBER (Fig. 3). This result was consistent across the study regions.

### Financial performance of the management scenarios

**Figure 4:**
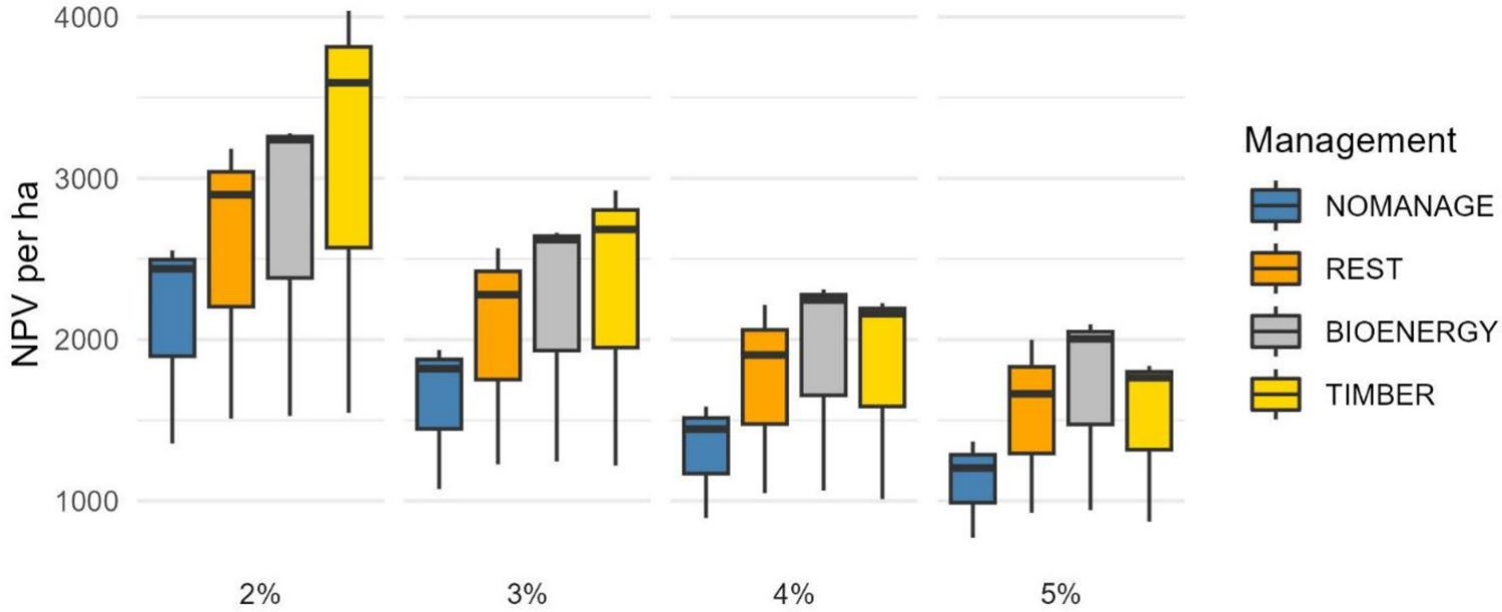
Net present values (NPV) € ha^−1^ associated with each management scenario. A box shows the first quartile to the third quartile value. The line inside the box represents the median, whereas the whisker shows the minimum and maximum NPVs associated with each scenario. Interest rate 2–5%.

The lowest financial performer (in all regions) was NOMANAGE, regardless of the interest rate applied (Fig. 4). The best performer with low-interest rates (2% and 3%) was TIMBER, whereas with higher interest rates (4% and 5%) the best financial performer was BIOENERGY (Fig. 4). The poor financial performance of TIMBER with higher interest rates (4% and 5%) was due to the fact that the drained peatland sites in the study regions were on average less productive than common commercial peatland forests in Finland. This indicates weak responses to silvicultural actions and relatively low cutting removals during rotation. The lowest NPVs were in Salamajärvi region associated with the NOMANAGE scenario, ranging from 772 € ha^−1^ (interest rate 5%) to 1 356 € ha^−1^ (2%), while the highest NPVs were in Mujejärvi region ranging from 1 764 € ha^−1^ (5%) to 4 037 € ha^−1^ (2%) under TIMBER scenario (Fig. 4: whisker).

### Cost-impact performance at different management scenarios

**Figure 5:**
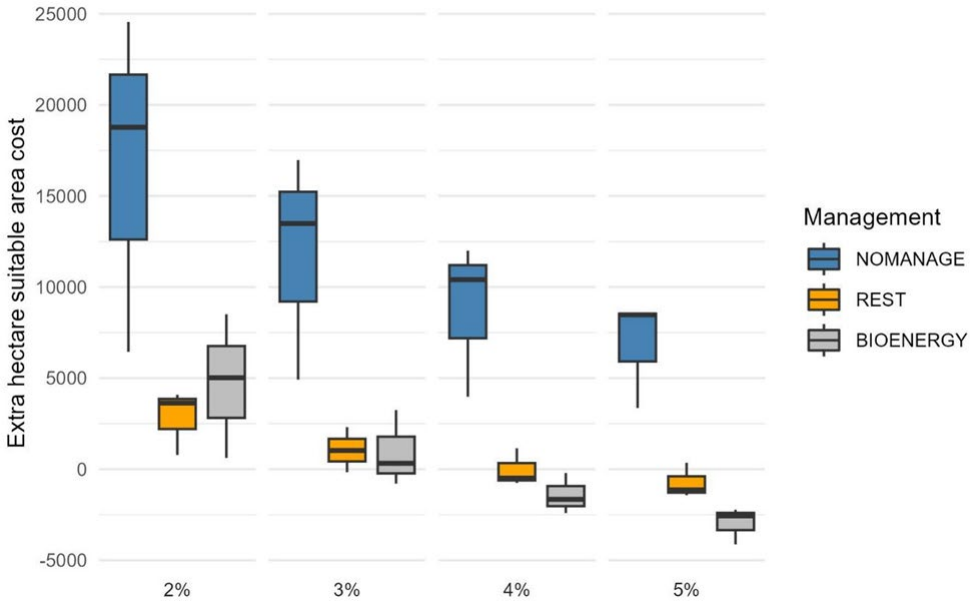
Median (line inside the box), minimum and maximum cost (whisker) of adding an extra hectare suitable for red-listed species, € ha^−1^. Minimum and maximum costs indicate cost-impact values for individual regions (Salamajärvi, Mujejärvi, or Olvassuo), while the box represents the first quartile to the third quartile value across the three study regions. Interest rate 2–5%. In calculating cost-impact values, TIMBER was set as the base scenario, and negative values indicate a *win*–*win* situation in relation to TIMBER.

The results showed that the REST and BIOENERGY scenarios were more cost-efficient to contribute habitat regeneration as indicted by red-listed plant species habitat potential modeling, than NOMANAGE (Fig. 5). For instance, with a 3% interest rate adding an extra hectare suitable for red-listed species cost on average 11 792 € ha^−1^ in NOMANAGE, while in REST and BIOENERGY, the cost was only 1 059 € ha^−1^ and 929 € ha^−1^, respectively (Fig. 5). The difference between NOMANAGE and the two other scenarios was, therefore, at least 11-fold. Further, with a 5% interest rate, the cost of an extra hectare suitable for red-listed species turned negative for both REST and BIOENERGY scenarios (Fig. 5). This is due to the fact that in TIMBER, the average NPV was lower than the average NPV of REST and BIOENERGY with a 5% interest rate (see Fig. 4), also turning the trade-off value negative. In practice, such a negative trade-off value indicates a *win–win* situation: higher NPV is produced simultaneously with enhanced biodiversity.

However, the variation of cost-impact values (expressed as € ha^−1^ for an extra hectare suitable for red-listed species) fluctuated widely: for instance, for the REST scenario, the highest cost-impact value with a 4% interest rate was 1 152 € ha^−1^ in Mujejärvi while the lowest cost-impact value was  − 751 € ha^−1^ in Salamajärvi (Fig. 5). Further, with a 2% interest rate and for NOMANAGE, the cost-impact value fluctuated between 6 445 € ha^−1^ and 24 557 € ha^−1^ (Fig. 5), implying manyfold values between regions.

## Discussion

Peatland management planning faces challenges regarding contrasting targets set for the environment, society, and economy. Compromises may therefore be needed to find the most cost-efficient strategy to reach the targets. A study by Juutinen et al.^51^ showed that a small decrease in NPV can increase biodiversity. Another study by Glenk et al.^43^ finds that early restoration action generates considerable financial benefits and creates increased resilience of peatlands under future climate change in Scotland. Makrickas et al.^74^ demonstrated that peatland forest rewetting can increase the economic value of a natural peatland forests while drainage of peatland forests for wood production has caused a significant economic loss in Lithuania. In many cases, landowners must consider which trade-offs between biodiversity and the economy are acceptable according to their management objectives. In this paper, we point out that also *win*–*win* situations are possible (33% probability), where biodiversity and economic benefits go hand in hand.

Our study shows that restoration (REST) results into the largest potential area of suitable habitat for the red-listed peatland plant species, followed by the production of bioenergy (BIOENERGY) and not managing the stands at all (NOMANAGE). Enhanced timber production (TIMBER) produces the smallest potential area of suitable habitat. This result is expected because the explanatory environmental variables, especially the proportion of undrained peatland area, were changed towards the desired direction. Therefore, the potential area of suitable habitat behaves similar way to environmental variables. In contrast, TIMBER resulted into the largest drainage area.

What is more interesting is that the difference between the four scenarios is consistent across the three study regions, which were highly different in terms of their forest stand structure, site fertility, and drainage percentage. This consistency indicates the robustness of our models to tackle the relevant parameters behind habitat area suitability. The proportion of undrained peatland area is one of the most influential variables in explaining the suitable habitat area for the red-listed species, as these plants are sensitive to drainage^25,75^. A larger undrained area corresponds to a greater potential suitable habitat for peatland plant species. This phenomenon is intricately linked to the restoration process of drained peatlands, which triggers a series of beneficial changes. Restoration activities lead to a gradual rise in the water table, the recovery of nutrient cycling, and shifts in plant communities toward wetter conditions^76^. These collective improvements have a profoundly positive effect on peatland ecosystems, facilitating the revival and expansion of vital plant species^22,23,76^. However, the empirical relationship between restoration measures and the revival of red-listed plant species is far more complex than generalist species. The uncertainties associated with the benefit of restoration measures to the red-listed species are profound which often require more targeted and specialized interventions for successful revival^10,76,77^. Another important variable, the volume of tree stand, correlates with the depth of the water table^78,79^. In this manner, we could predict, for example, that tree harvesting raises the water table. Hence, both the increase of undrained area and the rise of the water table in the drained area were simulated in the model, which together had a positive effect on the suitable habitat of the red-listed plant species.

While this study offers valuable insights into the impacts of different peatland management scenarios on biodiversity and financial performance, it's essential to acknowledge certain limitations. Firstly, the selection of four primary management scenarios—NOMANAGE, REST, BIOENERGY, and TIMBER—represents a significant step in understanding the trade-offs and synergies between biodiversity and economic gains. However, it's worth noting that there may exist other unexplored scenarios or variations in management practices that could provide additional perspectives. Secondly, the assumption of a 100-year timeframe for assessing the impacts of these scenarios is reasonable but may not fully capture the intricacies of long-term ecological changes, which can be highly complex and influenced by various factors^63^. Thirdly, the simulations of environmental variables introduce a level of uncertainty, such as climate-related variables, can be challenging to predict accurately over long timeframes^54^. In addition, while creating assumptions for the proportion of drained and undrained peatland areas, we used a simple and straightforward assumption due to limited data on how the ditches’ condition developed in the following 100 years. Although the data is scarce for old ditches, the filling in process of the ditches tends to slow down after 40 years^69^, which supports our assumption that some of the ditches could have draining function still after 100 years. Lastly, the generalizability of findings to other regions or ecosystems should be considered cautiously, given that this study focuses on specific landscapes in Finland. These limitations, while acknowledged, do not diminish the significance of the study's findings but rather provide avenues for future research and considerations for policy implications.

Although the NPV criterion is generally considered superior and is widely used^80^, there are also some disadvantages related to the methodology. Perhaps the most decisive disadvantage is related to how to value money flows (both costs and revenues) in the far distant future (in this study up to 100 years), i.e., what discount rate to be used? Recent forest economics literature suggests applying time declining discount rates^81,82^, but the fundamental question still remains—one has to choose a value or a function which is based on argumentation. In this study a constant discount rate of 2%, 3%, 4% or 5% was applied. This range (from 2 to 5%) covers the discount rates, particularly related to cases with long-horizon rotations^83^. Another disadvantage related to the NPV criterion is that it requires detailed information on all costs and revenues during the time horizon in question^84^. In this study all costs and revenues were based on simulations according to the four management scenarios indicating that they were included also in the NPV calculation as well.

REST and BIOENERGY scenarios were the most cost-efficient scenarios in increasing the potential area of suitable for red-listed species. With high-interest rates (4% and 5%), REST and BIOENERGY even enabled *win*–*win* conditions in terms of simultaneous financial gains (i.e., higher NPVs compared to TIMBER) and enhanced biodiversity (increased suitable habitat area compared to TIMBER). Since the management intervention in the REST and BIOENERGY scenarios is less intense than TIMBER, the cost of silvicultural treatment will be lower as well. REST and BIOENERGY scenarios also gain revenue from harvested trees. Therefore, with harvesting revenue and small silvicultural cost, REST and BIOENERGY scenarios will produce greater NPV than NOMANAGE (0 revenue) and TIMBER (high revenue, high cost).

Our result supports the findings of previous studies that peatland restoration can be cost-efficient^74,85^ and that the benefits can exceed the cost^49^. This finding is important to encourage landowners to consider peatland biodiversity in the management of their peatlands. It is, however, important to identify to which sites a particular management option should be assigned^41^. The restoration should be targeted to sites which have high potential species diversity or are located close to protected areas. In contrast, it may not be a good option to assign restoration and energy wood harvesting to sites which are profitable for timber production. Hence, there are tradeoffs to be considered, but the cost-impact approach could be used at the site level to identify cost-effective sites. It could also be linked to a participatory planning process^86^. The direct capital costs of restoration, caused by ditch blocking and removing trees from ditch lines, probably also depend on site characteristics, but we were not able to take this into account because the necessary information was not available. This is an interesting topic for future research.

In this study, we especially focused on drained peatlands, which have initially been drained for forestry. Just as in Finland, drained peatlands are also found in other countries, such as Sweden, the Baltic countries, and the Russian Federation. The developed approach can be utilized widely in different ecosystems when there is a need to reconsider peatland management options with fulfilling the needs of bioeconomy development, bioenergy production, and biodiversity protection. By conducting a comprehensive analysis of management options at three landscapes in Finland, the study identified cost-efficient management options that maximize ecological benefits while minimizing financial burden. These findings hold significant promise for enhancing the affordability and accessibility of EU restoration efforts, ultimately facilitating more sustainable land use practices and contributing to biodiversity conservation goals.

## Conclusion

Peatland management alternatives can be evaluated on a case-by-case basis based on our study findings, which can serve as a benchmark for ecological, financial, and cost-impact assessments. Three significant conclusions can be drawn. First, our findings indicate that different management alternatives do matter in terms of enhancing red-listed peatland plant species. Second, substantial cost savings can be achieved when choosing wisely the strategy to manage peatlands. Last, even a *win*–*win* condition is possible, in which higher NPV is achieved simultaneously with improved habitat suitability. In the future, it would be interesting to expand this framework to cover other non-marketed ecosystem services than biodiversity and further to analyze whether marketed costs (in the form of income losses of timber production) increase in line with e.g., an increase in carbon sequestration or berry picking or would there even be some cost-efficient synergy attainable for ecosystem services. Overall, our analysis can help evaluate the potential performance of alternative peatland management plans and to support the achievement of the EU’s biodiversity conservation targets in a cost-efficient manner.

### Supplementary Information

Supplementary Information.

## Data Availability

Data can be obtained from the corresponding author upon request.
