# Supplementary material for: Cost-efficient management of peatland to enhance biodiversity in Finland
Source: Sci Rep. 2024 Jan 30;14:2489. doi: 10.1038/s41598-024-52964-x (PMC10827728; doi:10.1038/s41598-024-52964-x)
Supplement: Supplementary file 1 — Supplementary Information. [file 41598_2024_52964_MOESM1_ESM.docx]

**Supplementary**

Table S1. Stumpage prices and silvicultural costs (in real terms) applied in assessing the NPV for NOMANAGE, REST, BIOENERGY, and TIMBER management scenarios.

| Stumpage prices | Sawlogs, € m^-3^ | | Pulpwood, € m^-3^ | Energywood, € m^-3^ |
| --- | --- | --- | --- | --- |
| First commercial thinning | pine: 40.62  spruce: 42.54  birch: 33.42 | | 12.36  12.07  12.09 |  |
| Intermediate thinnings | 49.86  50.98  38.50 | | 15.87  16.24  15.19 |  |
| Harvesting | 58.83  60.39  45.72 | | 18.88  20.05  18.12 |  |
| All cutting (thinning and harvesting) and tree species |  | |  | 3.50 for stems, crowns and stumps |
| Silvicultural costs | | | | |
| Planting | | 638.4 € ha^-1^ | | |
| Seeding | | 271.8 € ha^-1^ | | |
| Mounding | | 411.5 € ha^-1^ | | |
| Disc trenching | | 226.8 € ha^-1^ | | |
| Patch scarification | | 354.8 € ha^-1^ | | |
| Early cleaning | | 35 € hour^-1^ **^a)^** | | |
| Precommercial thinning | | 35 € hour^-1^ | | |
| Clearing of a thinning area | | 35 € hour^-1^ | | |
| Ditch network maintenance | | 223.3 € ha^-1^ | | |
| Fertilization | | 397.9 € ha^-1^ | | |

**^a)^**  Unit cost per hour, total costs calculated with time consumption models resulting in € ha^-1^

**Table S2.** Ditch spacing and depth were used in the SUSI simulator for each management scenario.

| Management scenario | | Undrained peatlands | Drained peatlands | Mineral soil stands ^a^) |
| --- | --- | --- | --- | --- |
|  |  |  |  |  |
| NOMANAGE | Ditch spacing | 150 m | 40 m | 150 m |
|  | Ditch depth | 0.1 m | 0.5 m at the beginning, shallower over time | 0.1 m and 0.5 m |
| REST | Ditch spacing | 150 m | 150 m | 150 m |
|  | Ditch depth | 0.1 m | 0.1 m | 0.1 m and 0.5 m |
| BIOENERGY | Ditch spacing | 150 m | 40 m | 150 m |
|  | Ditch depth | 0.1 m | 0.5 m at the beginning, shallower over time | 0.1 m and 0.5 m |
| TIMBER | Ditch spacing | 150 m | 40 m | 150 m |
|  | Ditch depth | 0.1 m | 0.7 m at the beginning, shallower over time; 0.9 m in ditch network maintenance* | 0.1 m and 0.5 m |

*^)^ No ditch network maintenance in the stands located in protected area.

^a)^ Mineral soil stands are typically undrained. However, the parameterization in SUSI model needs ditches; 0.1 m ditch in one end of the simulation domain (width 150 m), and 0.5 m in the other end.

**Table S3.** Share of drained peatland area (%) of those forest stands originally classified as "drained peatlands".

| YEAR | NOMANAGE | REST | BIOENERGY | TIMBER |
| --- | --- | --- | --- | --- |
| 0 | 100 | 0 | 100 | 100 |
| 5 | 96.5 | 0 | 96.5 | 100 |
| 10 | 93 | 0 | 93 | 100 |
| 15 | 89.5 | 0 | 89.5 | 100 |
| 20 | 86 | 0 | 86 | 100 |
| 25 | 82.5 | 0 | 82.5 | 100 |
| 30 | 79 | 0 | 79 | 100 |
| 35 | 75.5 | 0 | 75.5 | 100 |
| 40 | 72 | 0 | 72 | 100 |
| 45 | 68.5 | 0 | 68.5 | 100 |
| 50 | 65 | 0 | 65 | 100 |
| 55 | 61.5 | 0 | 61.5 | 100 |
| 60 | 58 | 0 | 58 | 100 |
| 65 | 54.5 | 0 | 54.5 | 100 |
| 70 | 51 | 0 | 51 | 100 |
| 75 | 47.5 | 0 | 47.5 | 100 |
| 80 | 44 | 0 | 44 | 100 |
| 85 | 40.5 | 0 | 40.5 | 100 |
| 90 | 37 | 0 | 37 | 100 |
| 95 | 33.5 | 0 | 33.5 | 100 |
| 100 | 30 | 0 | 30 | 100 |

**Table S4.** Share of undrained peatland area (%) of those forest stands originally classified as "drained peatlands".

| YEAR | NOMANAGE | REST | BIOENERGY | TIMBER |
| --- | --- | --- | --- | --- |
| 0 | 0 | 100 | 0 | 0 |
| 5 | 3.5 | 100 | 3.5 | 0 |
| 10 | 7 | 100 | 7 | 0 |
| 15 | 10.5 | 100 | 10.5 | 0 |
| 20 | 14 | 100 | 14 | 0 |
| 25 | 17.5 | 100 | 17.5 | 0 |
| 30 | 21 | 100 | 21 | 0 |
| 35 | 24.5 | 100 | 24.5 | 0 |
| 40 | 28 | 100 | 28 | 0 |
| 45 | 31.5 | 100 | 31.5 | 0 |
| 50 | 35 | 100 | 35 | 0 |
| 55 | 38.5 | 100 | 38.5 | 0 |
| 60 | 42 | 100 | 42 | 0 |
| 65 | 45.5 | 100 | 45.5 | 0 |
| 70 | 49 | 100 | 49 | 0 |
| 75 | 52.5 | 100 | 52.5 | 0 |
| 80 | 56 | 100 | 56 | 0 |
| 85 | 59.5 | 100 | 59.5 | 0 |
| 90 | 63 | 100 | 63 | 0 |
| 95 | 66.5 | 100 | 66.5 | 0 |
| 100 | 70 | 100 | 70 | 0 |
